# Supplementary material for: Sustained-input switches for transcription factors and microRNAs are central building blocks of eukaryotic gene circuits
Source: Genome Biol. 2013 Aug 23;14(8):R85. doi: 10.1186/gb-2013-14-8-r85 (PMC4054853; doi:10.1186/gb-2013-14-8-r85)
Supplement: Additional file 5 — HTML Browsable Motif Output. Zipped folder containing all WaRSwap and FANMOD motif output, viewable in a web browser. [file gb-2013-14-8-r85-S5.ZIP › HTML_browsable_motif_output/WaRSwap_ath_TwoLoops_withSelfLoops/sigs_WSWRSLDraw_TAIR10_TwoLoops_SLColors-2500.pvals.heatmaps.html/motif_id_SelfLoops_tftype_ath_upstream_-3000_0.html]

```
BG_MODEL = WARSwap
MOTIF_ID = SelfLoops_tftype
TF_TYPE = upstream
UPSTREAM = 0_


PVals
FNR = 0.2	FNR = 0.4	FNR = 0.6	FNR = 0.8
deltaG = 60	0.001	0.001	0.004	0.186
deltaG = 70	0	0	0.004	0.19
deltaG = 80	0	0	0.003	0.177

ZScores
FNR = 0.2	FNR = 0.4	FNR = 0.6	FNR = 0.8
deltaG = 60	3.187	2.944	2.486	0.505
deltaG = 70	3.198	2.97	2.546	0.493
deltaG = 80	3.188	2.957	2.517	0.504

StDevs
FNR = 0.2	FNR = 0.4	FNR = 0.6	FNR = 0.8
deltaG = 60	1.967	2.157	1.909	1.372
deltaG = 70	1.97	2.144	1.874	1.352
deltaG = 80	1.977	2.137	1.886	1.366
```
